# Severe Congenital Toxoplasmosis: A Case Report and Strain Characterization

**DOI:** 10.1155/2015/851085

**Published:** 2015-01-18

**Authors:** Bahador Sarkari, Samaneh Abdolahi Khabisi

**Affiliations:** ^1^Basic Sciences in Infectious Diseases Research Center, Shiraz University of Medical Sciences, Shiraz, Iran; ^2^Department of Parasitology and Mycology, School of Medicine, Shiraz University of Medical Sciences, Shiraz, Iran

## Abstract

We report a fatal congenital toxoplasmosis case in an Iranian woman in the south of Iran. A pregnant mother had been admitted at the 15th week of her pregnancy on account of a febrile illness, symptoms of common cold, and enlargement of submandibular lymph nodes. Serological testing of the mother's serum revealed positive IgG and IgM anti-*Toxoplasma* antibodies. Amniotic fluid was taken and evaluated by polymerase chain reaction (PCR) assay with a direct amplification of the *Toxoplasma* URPT gene which was found to be positive. Sequencing and analysis of PCR product revealed that the isolate has the most similarity with type I of *Toxoplasma gondii*. Fetal scan showed anomaly in fetus including mild hydrocephaly. Termination of the pregnancy was suggested by the physician and pregnancy was terminated 178 days after conception.

## 1. Introduction

*Toxoplasma gondii* is a protozoan with worldwide distribution [1]. While toxoplasmosis is a mild disease in immunocompetent individuals, the disease is severe and life threatening in immunocompromised patients [2]. The infection in immunocompromised individuals such as transplant recipients and HIV-positive patients can result in severe consequences including encephalitis, chorioretinitis, and myocarditis [2].

Congenital toxoplasmosis (CT) is the most serious manifestation of the disease, resulting from transplacental contamination of the fetus with* Toxoplasma gondii* during pregnancy [1, 3]. Severity of the disease mainly depends on the gestational age at transmission. Infection of fetus at the first trimester of pregnancy may cause severe damages to the fetus whereas the fetal disease is less severe in the later trimesters. Infections at the early gestational age may cause anemia, jaundice, chorioretinitis, seizure, and hydrocephalus of fetus. Late sequels of congenital toxoplasmosis are sensorineural deafness, microcephaly, mental retardation, visual defect, and developmental delay. Here we report a case of severe congenital toxoplasmosis in an infant. The strain that caused the infection was somewhat characterized.

## 2. Case

The case was an Iranian woman, aged 25, married at age of 24, and had pregnancy with her first baby six months after marriage. She had been admitted to an outpatient clinic at the 15th week of her pregnancy on account of a febrile illness, headache, enlargement of lymph nodes, and symptoms of common cold. The patient was given a one-week course of cefixime (400 mg/day) with Co-amoxiclav (625 mg tablet every 8 hrs) but remained symptomatic despite receiving this treatment. At second admission to an ear, nose, and throat (ENT) specialist,* Toxoplasma* serological tests, both IgG and IgM, were requested for the patient. Both tests were found to be strongly positive (IgG 98 IU/mL, reference positive range > 8 IU/mL; IgM 44 IU/mL, reference positive range >10; Monobind ELISA kit). Serum samples have been sent to a reference laboratory one week later and the results were further confirmed. C-reactive protein (CRP) and mono tests were both negative. Elevated erythrocyte sedimentation rate (ESR) was noticed in her hematology tests. The rest of biochemistry and hematological indices were normal. Fine-needle aspiration biopsy (FNA) of neck soft tissue revealed a few lymphadenopathies in left submandibular region with decreased echogenic hilum. The largest lymph nodes were seen in diameters of 20 × 7 mm and 22 × 10 mm.* T. gondii* parasites were not seen in the lymph node biopsy specimen with use of conventional histologic stains.

Fetal contamination was investigated by ultrasonography (U/S) and by amniotic fluid analysis. Results of U/S at this stage (17th week) showed that the fetus is alive with no gross anomaly. Ten milliliters of clear amniotic fluid was aspirated and sent to laboratory for molecular testing. PCR of amniotic fluid was positive. The patient was given a course of spiramycin at a dose of 1 g orally, every 8 hours, followed by pyrimethamine (50 mg/day orally) and sulfadiazine (3 g/day orally in 2-3 divided doses). The patient could not tolerate the pyrimethamine-sulfadiazine regimen and the treatment was terminated. At 24th week of pregnancy, fetal scan showed anomaly in fetus including mild hydrocephaly and decreased amniotic fluid. Termination of the pregnancy was suggested by the physician. Pregnancy was terminated at 178 days after conception. The fetus (male) was covered with meconium, fetal organs being grossly autolysed. The skin of the trunk was dark with many petechial purpura, same as what was seen in the skin of his mother due to drug reaction.

The patient was housekeeper and used to eat meat, especially kebab. She had also contact with cats and lives in a farming area.

DNA extraction and PCR on amniotic fluid: DNA was extracted from the amniotic fluid. Extraction was done using 8 *μ*L of proteinase K (20 mg/mL) and 250 *μ*L of lysis buffer (50 mL of Tris-HCl (100 Mm), pH = 8; 1 mM of EDTA, pH = 8.0; 1% Tween 20) followed by phenol/chloroform/isoamyl extraction. Absolute ethanol was used to precipitate the DNA. Precipitated DNA was resuspended in 100 *μ*L of double-distilled water and stored at 4°C until use. PCR primer sets were used for amplifying fragments of the UPRT (uracil phosphoribosyltransferase) gene of* T. gondii*. PCR was done using UPRT3 (forward: 5′-ACTGCGACGACATACTGGAGAAC-3′) and UPRT4 (reverse: 5′-AAGAAAACAAAGCGGAACAACAA-3′) primers. PCR reaction was performed in a total volume of 25 *μ*L containing 5 *μ*L of DNA template, 1 *μ*L of dNTP (0.2 mM), 1 *μ*L of MgCl_2_ (1.5 Mm), 0.5 *μ*L of Taq DNA polymerase (2.5 U/100 *μ*L), 0.5 *μ*L of each primer (10 Pmol), and 2.5 *μ*L of PCR buffer (10x). Thermocycler was programmed by one cycle of initial denaturation at 94°C for 2 min, followed by 30 cycles of denaturation at 94°C for 1 min, annealing at 60°C for 2 min, and extension at 72°C for 3 min and final extension at 94°C for 1 min. PCR product was separated by electrophoresis in 1.5% agarose gel and stained with ethidium bromide.

**Figure 1:**
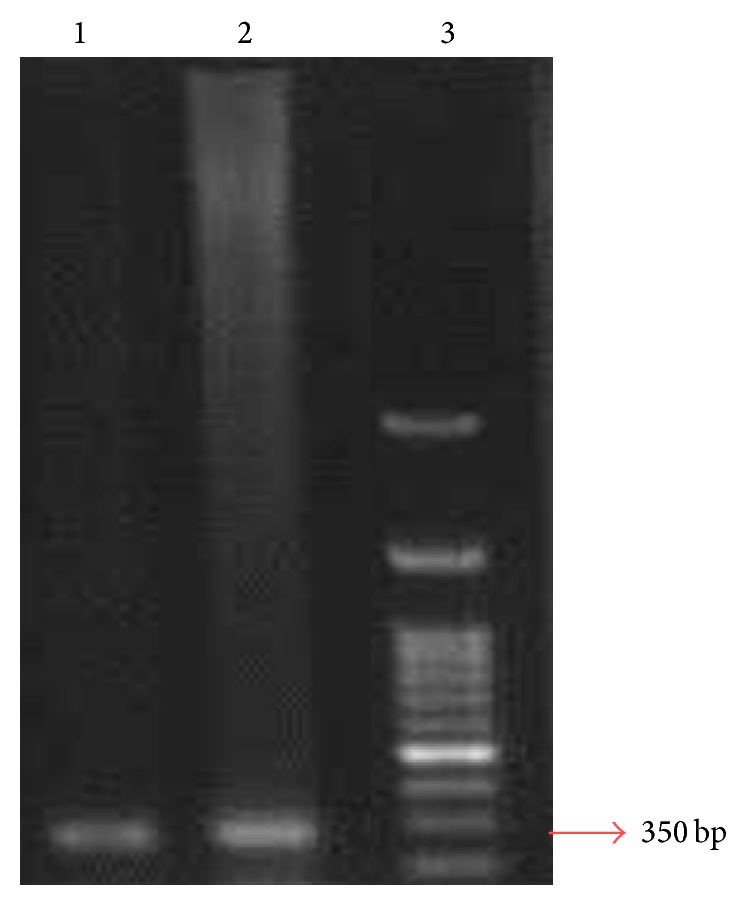
PCR product of* Toxoplasma gondii* amplified by UPRT primer. Lane 1: positive control (strain Rh of* Toxoplasma gondii* (type I)), lane 2: patient's amniotic sample, and lane 3: molecular marker.

After PCR amplification, an approximately 350 bp PCR product representing a fragment of the UPRT gene was amplified (Figure 1).

PCR product was excised from 1.5% agarose gel and purified with a DNA Gel Extraction Kit (Bioneer's AccuPrep Gel Purification Kit), according to the manufacturer's instructions. Purified PCR product was sequenced, using the same primers as described for the amplification process. BLAST analysis was used to compare the sequence with those of available* T. gondii* sequences in the GenBank.

**Figure 2:**
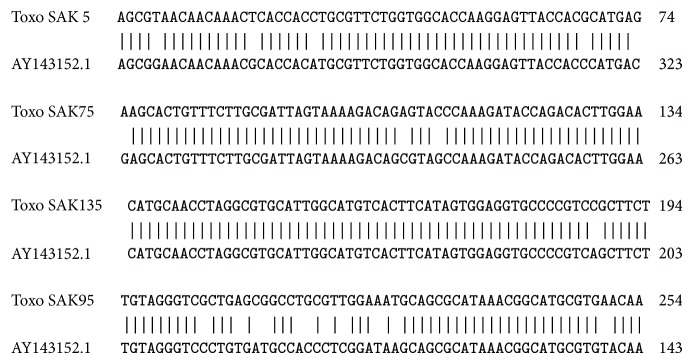
Alignment of sequence of* Toxoplasma* isolated from amniotic fluid of the patient. Toxo SAK: sample from the patient; AY143152.1: sequence ID of* Toxoplasma gondii* strain type I uracil phosphoribosyltransferase (UPRT) gene.

The sequence analysis demonstrated that the case has 92% identity with those of available sequences for type I* T. gondii* in GenBank (Figure 2).

## 3. Discussion

*Toxoplasma gondii* infection during pregnancy may result in fetus infection in about 30% of cases [1]. During the third trimester of pregnancy, the transmission rate increases from 30% at 6 months of pregnancy to 100% during the last weeks [1]. However, third-trimester congenital infections appear to be mild and rarely result in a severely affected infant.

Prenatal diagnosis of CT relies commonly on the molecular based detection of* Toxoplasma gondii* DNA. Here we reported a severe case of congenital toxoplasmosis which resulted in termination of pregnancy. The isolate has the most similarity with type I of* Toxoplasma gondii.* In Europe, type II genotype circulates in humans and this type is related to acquired and also congenital toxoplasmosis [4]. In a study by Ajzenberg et al., genotype of 86* T. gondii* isolates collected from patients with congenital toxoplasmosis and their association with clinical findings were evaluated. Result of the study demonstrated that type II isolates are the largely predominant type and type I and atypical isolates were not found in asymptomatic or benign congenital toxoplasmosis [4]. Genotype analysis of* Toxoplasma* strains in Spain revealed that* T. gondii* type II were the most prevalent (52%) genotype in immunocompromised patients, whereas strains of type I were present in 75% of the congenital infection cases. This is rather different from previous study in the region and also the study in France which showed that type II strains were mostly associated with all kinds of human toxoplasmosis. Effects of selection in the process of culturing and isolation, prior to strain characterization, have been accounted for by these differences [5].

Type I genotype is considered to be the most virulent type, with a high level of parasitemia [6]. This may cause an increase in the risk of transplacental transmission, producing severe symptoms in the fetus or newborn. Findings of the current case report are in keeping with this concept.

In this study PCR has been used for detection of* Toxoplasma* DNA in amniotic fluid of the patient. This procedure has a relatively high sensitivity and almost 100 percent specificity [7]. A PCR negative test on amniotic fluid does not rule out the infection but a positive result guarantees the infection of the amniotic fluid.

Taken together, findings of this study are important as they will provide a better understanding of congenital toxoplasmosis and its outcome and also possible association between parasite genotypes and congenital infection. This study also highlighted the lack of efficient medication for treatment of toxoplasmosis during pregnancy.
